# Nomograms based on inflammatory biomarkers for predicting tumor grade and micro-vascular invasion in stage I/II hepatocellular carcinoma

**DOI:** 10.1042/BSR20180464

**Published:** 2018-11-14

**Authors:** Peng Li, Wei Huang, Feng Wang, Ye-Fang Ke, Lin Gao, Ke-Qing Shi, Meng-Tao Zhou, Bi-Cheng Chen

**Affiliations:** 1 Department of Pathology, The First Affiliated Hospital of Wenzhou Medical University, Wenzhou, China; 2 Key Laboratory of Diagnosis and Treatment of Severe Hepato-Pancreatic Diseases of Zhejiang Province, The First Affiliated Hospital of Wenzhou Medical University, Wenzhou, China; 3 Precision Medical Center Laboratory, The First Affiliated Hospital of Wenzhou Medical University, Wenzhou, China

**Keywords:** hepatocellular carcinoma, inflammatory biomarkers, micro-vascular invasion, nomogram, tumor grade

## Abstract

**Background:** Increasing evidences reveal that inflammation plays a critical role in tumorigenesis and progression. We aimed to develop the nomograms based on inflammatory biomarkers to predict micro-vascular invasion (MVI) and tumor grade in stage I/II hepatocellular carcinoma (HCC).

**Methods:** A retrospective cohort of 627 patients with stage I/II HCC between January 2007 and December 2014 was included in the study. Logistic regression was performed to identify the independent risk factors of tumor grade and MVI. The significant predictors including neutrophil-to-lymphocyte ratio (NLR), derived neutrophil-to-lymphocyte ratio (dNLR), lymphocyte-to-monocyte ratio (LMR), tumor volume age, and tumor size were subsequently incorporated to build the nomograms. The prediction accuracies of the nomograms were evaluated using the area under the receiver operating characteristic (ROC) curve.

**Results:** The independent risk factors for tumor grade were NLR, dNLR, and tumor volume (*P*<0.001, *P*=0.001, and *P*<0.001, respectively), which were assembled into tumor grade nomogram. MVI nomogram was developed by dNLR, LMR, age, and tumor size (*P*<0.001, *P*<0.001, *P*<0.001, and *P*=0.001, respectively) which were the independent predictors for MVI. The area under the ROC curve of nomograms for predicting tumor grade and MVI were 0.727 (95% confidence intervals [CI]: 0.690–0.761) and 0.839 (95% CI: 0.808–0.867), respectively. Patients who had a nomogram score of less than 100 and 79 were considered to have high possibility of moderate grade and have low risks of MVI presence, respectively.

**Conclusion:** We successfully developed nomograms predicting tumor grade and MVI based on inflammatory biomarkers with high accuracy, leading to a rational therapeutic choice for stage I/II HCC.

## Introduction

Hepatocellular carcinoma (HCC) is the sixth most commonly diagnosed cancer worldwide and ranks as the second most common cause of cancer death [1]. Chronic infection with hepatitis B or C virus, long-term exposure to alcohol or aflatoxin, or non-alcoholic fatty liver disease are well-defined risk factors that can result in the development of HCC [2]. Hepatic resection and transplantation are still the mainstay of treatment in chosen patients [3]. Histopathologic grade of differentiation and micro-vascular invasion (MVI) are regarded as the two most important prognostic factors of tumor histological features, which strongly influence not only tumor recurrence but also patient survival [4,5]. MVI of a tumor is a prior condition for a dissemination or possible implantation of malignant cells and is not restricted to advanced tumor stages. An accurate preoperative estimation of tumor grade and the presence of MVI can help choose suitable surgical methods for patients based on risk-benefit evaluation. Patients with the early T stage are the primary candidates for curative treatments such as liver resection or transplantation [3], thus it has specific clinical significance to perform an assessment of tumor grade and MVI in the early stage HCC.

Currently, many efforts on preoperative estimation of MVI and tumor grade have been made over the past decade [6]. Morphological characteristics of the tumor, including tumor size and number, as well as tumor biomarkers are the factors that were most constantly found to estimate the risk of both tumor grade and vascular invasion [4,7–9]. However, the lack of prospective confirmation and the potential inter-observer variability make these results difficult to account. These serum markers can also be abnormally high with advanced fibrosis without HCC.

Recent studies have demonstrated that cancer progression and prognosis are associated with not only tumor characteristics but also the host inflammatory response [10–12]. In the clinical setting, levels of factors, such as neutrophils, lymphocytes, platelets, and C-reactive protein, were considered as representation of inflammatory reaction. Interestingly, several studies demonstrated that a series of combination of these parameters, such as neutrophil-to-lymphocyte ratio (NLR), platelet-to-lymphocyte ratio (PLR), lymphocyte-to-monocyte ratio (LMR), and derived neutrophil-to-lymphocyte ratio (dNLR) were significantly associated with the prognosis in various cancers, including HCC [13–17]. However, few previous studies discussed these inflammatory biomarkers comprehensively to evaluate their prognostic function on tumor grade and MVI.

The model of nomogram can provide an individualized, evidence-based, and highly accurate risk assessment. Many studies have shown that the development of nomograms results in a successful prediction of tumor prognosis. The objective of the current study was to develop nomograms based on inflammatory biomarkers for tumor grade and MVI in patients with stage I/II HCC.

## Methods

### Patients

A cohort of patients with HCC from the First Affiliated Hospital of Wenzhou Medical University between January 2007 and December 2014 were enrolled, retrospectively. The study was approved by the Institutional Ethics Committee of the First Affiliated Hospital of Wenzhou Medical University. Informed consent was obtained from all patients for their data to be used for study.

The inclusion criteria were: (1) patients were aged 18 years or older; (2) underwent surgical resection; (3) pathological diagnosis of HCC; (4) HCC of stage I/II according to the 7th Edition of the TNM-UICC/AJCC classification [18]: T_1-2_ N_0_ M_0_ (T1: single tumor without vascular invasion; T2: single tumor with vascular invasion, or multiple tumors, none >5 cm); and (5) laboratory tests were obtained before surgery. The exclusion criteria were as follows: (1) patients with hematologic disorder, other malignant diseases, human immunodeficiency virus, infections, and hyperpyrexia; (2) patients who had previously taken anti-inflammatory medicines or received immunosuppressive therapy including recent steroid exposure, or with chronic inflammatory diseases including infections and autoimmune diseases; (3) patients received any preoperative anti-tumor therapy; and (4) patients that did not have complete clinical and pathological data.

### Clinicopathologic variables

Patients’ demographic and clinicopathological variables, including age, sex, tumor grade, MVI, number of tumor nodules, pathogenesis, and cirrhosis based on contrast-enhanced MRI [19] etc., were retrieved from the database. Both tumor grade and presence of MVI were identified by experienced pathologists. Tumor grade was scored using the modified nuclear grading system summarized by Edmondson and Steiner [20–22]; specifically, modified Edmondson–Steiner grades 1/2 were defined as well-differentiated, and grades 3/4 as moderately/poorly differentiated. The definition of MVI was in line with that reported by Roayaie et al. [23,24]. Briefly, MVI was defined as the presence of tumor in a portal vein, hepatic vein, or a large capsular vessel of the surrounding hepatic tissue lined by endothelium that was visible only on microscopy. Routine examination included blood routine examination, liver function tests, blood coagulation function, serum α-fetoprotein level, and body mass index (BMI). The neutrophil, lymphocyte, monocyte, and platelet counts were retrieved from blood routine test.

### Statistical analysis

Statistical evaluation was conducted with SPSS 22.0 (SPSS Inc., Chicago, IL, U.S.A.) and R 3.1.2 software (Institute for Statistics and Mathematics, Vienna, Austria). Continuous variables were expressed as mean ± S.D. and compared using the Student’s *t* tests. Categorical variables were compared using the χ^2^ test or Fisher’s exact test. The significance of each variable was assessed by univariate and multivariate logistic regression analysis for investigating the independent risk factors of tumor grade and presence of MVI. The tumor-related variables, including number, size, and volume were assessed by preoperative imaging studies. Tumor grade and MVI were based on postoperative histopathologic data. *P*<0.05 was considered statistically significant. All confidence intervals (CI) were stated at the 95% confidence level.

A nomogram was formulated based on the results of multivariate logistic regression analysis and by using R software. The nomogram is based on proportionally converting each regression coefficient in multivariate logistic regression to a 0- to 100-point scale. The effect of the variable with the highest β coefficient (absolute value) is assigned 100 points. The points are added across independent variables to derive total points, which are converted to the predicted probabilities.

The performance of nomogram was evaluated with a calibration curve in which predicted outcomes versus observed outcomes are graphically depicted, which made it possible to conduct further comparison of accuracy in estimating prognosis. For a well calibrated model, the predictions should fall on a 45° diagonal line. Given that all predictive nomograms tend to be over-fitted to the original sample, the model was validated internally using bootstrap re-sampling to assess for and quantify any overfitting. Receiver operating characteristic (ROC) curve analysis was used to calculate the optimal cutoff values that were determined by maximizing the Youden index (i.e., sensitivity + specificity − 1). Accuracy of the optimal cutoff value was assessed by the sensitivity, specificity, predictive values, and likelihood ratios.

## Results

### Clinicopathologic characteristics

**Figure 1:**
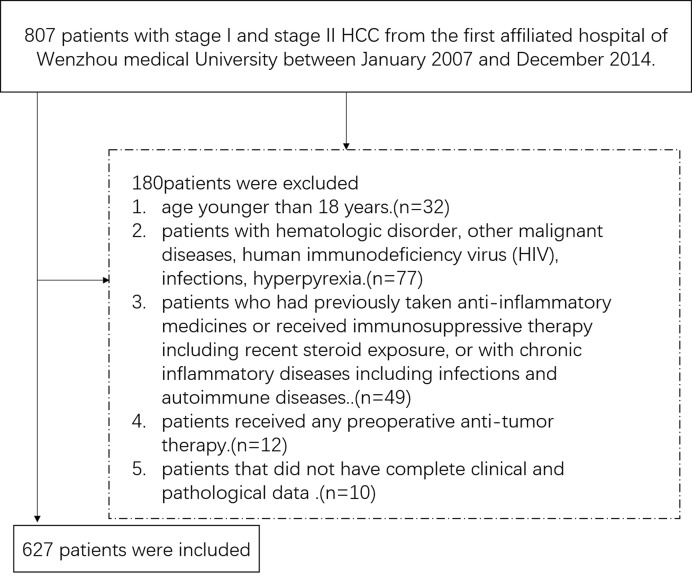
A flow diagram of study participants

During the study period, 627 patients with stage I and stage II HCC who met the inclusion criteria were enrolled (Figure 1). The descriptive clinicopathologic characteristics of the patients are listed in Table 1. The mean age was 56.00 years (range: 23.00–85.00). There were more male patients than female patients, with a male/female ratio of 5.6. Of all 627 included patients, 405 (64.6%) patients were with cirrhosis. The predominant etiologies of HCC were chronic infection with hepatitis B virus (HBV) and alcohol abuse. Most patients (512, 81.7%) were diagnosed as moderate differentiation of primary tumor, while 115 (18.3%) patients were diagnosed as poor differentiation. Histopathologically identified MVI was found in 174 (27.8%) patients in the cohort.

**Table 1:** Clinicopathologic features

Variable	All patients (*n*=627)
Age (year)	56.00 ± 11.59
BMI (kg/m^2^)	22.48 ± 3.86
Male sex, *n* (%)	532 (84.8%)
Neutrophil (*10^9^/l)	3.26 ± 1.49
Lymphocyte (*10^9^/l)	1.85 ± 0.81
Monocyte (*10^9^/l)	0.58 ± 0.31
NLR	2.14 ± 1.77
LMR	4.22 ± 3.56
PLR	91.35 ± 76.13
dNLR	3.05 ± 7.04
TB (mol/l)	1.13 ± 0.25
Albumin (g/l)	38.05 ± 4.88
ALT(µ/l)	53.92 ± 62.79
AST(µ/l)	63.16 ± 106.35
AKP(µ/l)	105.31 ± 82.36
GGT(µ/l)	97.38 ± 145.84
Creatinine (mg/dl)	0.78 ± 0.18
PT (s)	14.29 ± 1.94
PTA (%)	87.38 ± 14.46
INR	1.27 ± 3.89
WBC (*10^9^/l)	5.98 ± 4.61
Platelets (*10^9^/l)	136.96 ± 69.59
lg AFP (ng/ml)	1.95 ± 1.10
Tumor size	5.04 ± 3.33
Tumor volume (log10 cm^3^)	1.50 ± 0.84
Tumor number (*n*, %)	
1	524 (83.6%)
>1	103 (16.4%)
Cirrhosis (*n*, %)	
Yes	405 (64.6%)
No	222 (35.4%)
Tumor grade (*n*, %)	
1/2	512 (81.7%)
3/4	115 (18.3%)
MVI (*n*, %)	
Presence	174 (27.8%)
Absence	453 (72.2%)

Abbreviations: AKP, alkline phosphatase; ALT, alanine aminotransferase; AST, aspartate aminotransferase; GGT, γ-glutamyltransferase; INR, international normalized ratio; PT, prothrombin time; PTA, prothrombin activity; WBCs, white blood cells.

### Prognostic value of inflammatory biomarkers

The results of univariate analysis for tumor grade and the presence of MVI were presented in Table 2 and Supplementary Table S1. Univariate analysis showed that neutrophil (*P*<0.001), NLR (*P*<0.001), PLR (*P*=0.011), dNLR (*P*<0.001), tumor size (*P*<0.001), and tumor volume (*P*<0.001) were the independent risk factors of tumor grade. Age (*P*=0.001), neutrophil (*P*<0.001), monocyte (*P*<0.001), NLR (*P*<0.001), LMR (*P*<0.001), dNLR (*P*<0.001), total bilirubin (TB) (*P*=0.003), log (α-fetoprotein [AFP]) (*P*=0.004), tumor size (*P*<0.001), and log (tumor volume) (*P*=0.005) were significantly associated with MVI. Subsequently, all these significant factors above were entered into multivariate analysis to adjust the effects of covariates for tumor grade and the presence of MVI. As Table 3 showed, NLR (OR: 1.377, 95% CI: 1.218–1.556, *P*<0.001), dNLR (OR: 1.060, 95% CI: 1.026–1.096, *P*=0.001), tumor volume (OR: 1.773, 95% CI: 1.347–2.334, *P*<0.001), were independently associated with tumor grade, while dNLR (OR: 1.191; 95% CI: 1.126–1.261, *P*<0.001), LMR (OR: 0.382, 95% CI: 0.311–0.470, *P*<0.001); age (OR: 0.964, 95% CI: 0.946*–*0.983, *P*<0.001), tumor size (OR: 1.110, 95% CI: 1.042–1.183, *P*=0.001) were identified as independent predictors of MVI.

**Table 2:** Univariate logistic regression analysis of tumor grade, and tumor grade and MVI presence based on preoperative data

Variable	Tumor grade			MVI		
	OR	95% CI	*P*	OR	95% CI	*P*
Age (year)	1.000	0.983–1.018	0.967	0.975	0.960-0.990	0.001
BMI (kg/m^2^)	1.052	0.996–1.111	0.069	1.016	0.970-1.064	0.500
Male sex, *n* (%)	1.235	0.682–2.233	0.486	1.089	0.664–1.786	0.735
Neutrophil (10^9^/l)	1.642	1.429–1.887	<0.001	1.498	1.323–1.696	<0.001
Lymphocyte (10^9^/l)	1.004	0.783–1.287	0.978	0.932	0.750–1.157	0.521
Monocyte (10^9^/l)	1.329	0.712–2.480	0.372	524.6	190.11–1447.5	<0.001
NLR	1.441	1.279–1.623	<0.001	1.282	1.154–1.425	<0.001
LMR	0.943	0.877–1.014	0.111	0.470	0.402–0.551	<0.001
PLR	1.003	1.001–1.005	0.011	1.001	0.999–1.004	0.199
dNLR	1.070	1.035–1.106	<0.001	1.138	1.086–1.191	<0.001
TB (µmol/l)	1.948	0.899–4.221	0.091	2.854	1.443–5.643	0.003
Albumin (g/l)	1.007	0.966–1.050	0.751	1.011	0.976–1.049	0.538
ALT (µ/l)	0.999	0.996–1.003	0.661	1.001	0.998–1.003	0.616
AST (µ/l)	1.000	0.997–1.002	0.727	1.001	0.999–1.002	0.368
AKP (µ/l)	1.000	0.998–1.003	0.859	1.000	0.997–1.002	0.872
GGT (µ/l)	1.000	0.999–1.002	0.758	1.000	0.999–1.001	0.968
Creatinine (mg/dl)	0.431	0.132–1.410	0.164	0.597	0.218–1.635	0.16
PT (s)	0.968	0.858–1.093	0.603	0.993	0.905–1.090	0.883
PTA (%)	1.003	0.989–1.018	0.643	0.996	0.984–1.008	0.537
INR	1.057	0.918–1.217	0.441	0.955	0.722–1.264	0.749
WBC (10^9^/l)	1.052	0.993–1.113	0.083	1.048	0.993–1.107	0.086
Platelets (10^9^/l)	1.002	0.999–1.005	0.182	1.000	0.998–1.003	0.765
lg AFP (ng/ml)	1.121	0.933–1.347	0.223	1.267	1.079–1.487	0.004
Tumor size	1.129	1.608–1.193	<0.001	1.113	1.058–1.171	<0.001
Tumor volume (log10 cm^3^)	1.845	1.425–2.389	<0.001	1.356	1.095–1.680	0.005
Tumor number (*n*, %)	1.446	0.868–2.406	0.156	1.147	0.722–1.822	0.561
Cirrhosis (*n*, %)	1.034	0.677–1.581	0.877	1.310	0.902–1.903	0.157

Abbreviations: AKP, alkline phosphatase; ALT, alanine aminotransferase; AST, aspartate aminotransferase; GGT, γ-glutamyltransferase; INR, international normalized ratio; PT, prothrombin time; PTA, prothrombin activity; WBCs, white blood cells.

**Table 3:** Multivariate logistic regression analysis of independent risk factors predicting tumor grade and MVI

Variable	Tumor grade						MVI					
	OR	95% CI	Sensitivity	Specificity	Cutoff value	*P* value	OR	95% CI	Sensitivity	Specificity	Cutoff Value	*P* value
NLR	1.377	1.218–1.556	62.61	65.04	1.86	<0.001						
dNLR	1.060	1.026–1.096	40.87	83.01	2.56	0.001	1.191	1.126–1.261	42.53	88.08	2.61	<0.001
Tumor volume (log10 cm^3)^	1.773	1.347–2.334	53.91	72.27	1.82	<0.001						
LMR							0.382	0.311–0.470	72.41	80.13	2.57	<0.001
Age							0.964	0.946–0.983	37.93	75.06	50	<0.001
Tumor size							1.110	1.042–1.183	37.93	73.95	5	0.001

### Development of a predicting nomogram

**Figure 2:**
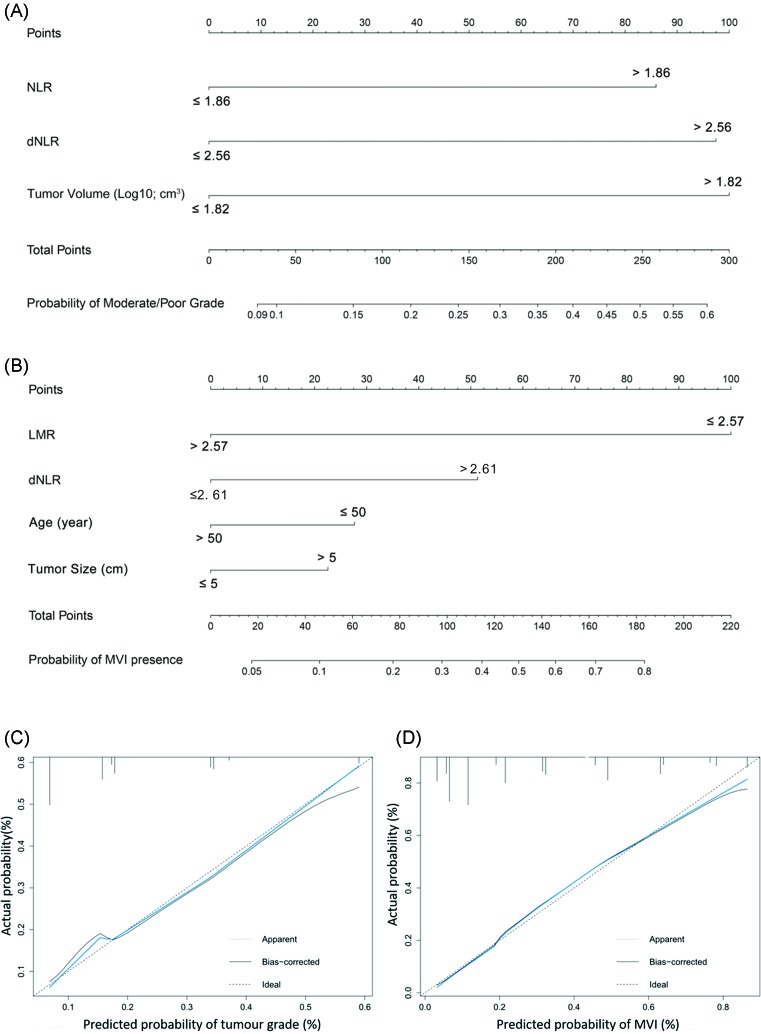
Nomogram to estimate tumor grade and MVI presence preoperatively in stage I/II HCC Nomograms can be interpreted by summing up the points assigned to each variable, which is indicated at the top of scale. The sum of these numbers is located on the total points axis and a line is drawn downwards to determine the tumor grade and MVI probabilities. (**A**) Nomogram for predicting tumor grade; (**B**) nomogram for predicting probability of MVI. Calibration plot of the nomogram for predicting the risk of tumor grade (**C**), and MVI presence (**D**) (bootstrap 1000 repetitions). The *x*-axis is nomogram-predicted probability and *y*-axis is actual probability. The reference line is 45° and indicates perfect calibration.

The significantly independent risk factors of tumor grade and MVI in multivariate logistic regression were incorporated into the tumor grade and MVI nomograms, respectively (Figure 2A,B). Nomograms can be described by summing up the points assigned to each variable, which was indicated at the top of scale. The total points could be converted to predict the probability of tumor grade and MVI for a patient in the lowest scale. Calibration plots graphically showed good agreement on the tumor grade and the presence of MVI between the risk estimation by the nomogram and histopathologic confirmation on surgical specimens (Figure 2C,D).

### Risk of tumor grade and MVI based on the nomogram scores

**Figure 3:**
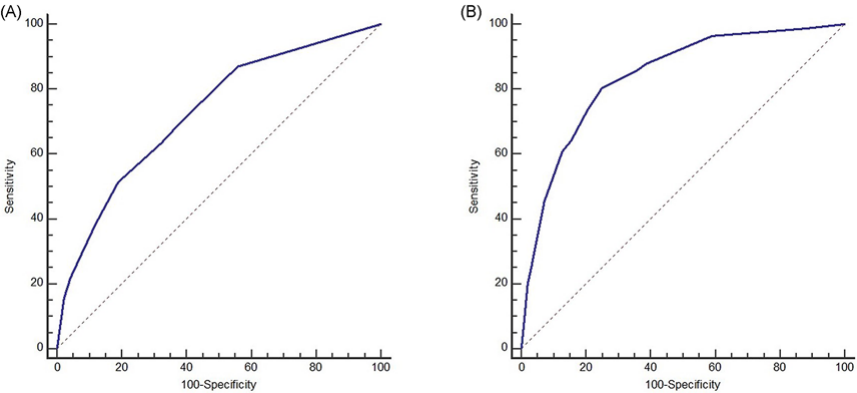
The accuracy of the nomogram for predicting moderate/poor grade and MVI using ROC curve (**A**) Accuracy for tumor grade nomogram. (**B**) Accuracy for MVI nomogram.

The optimal cutoff values of the total nomogram scores for tumor grade and MVI were determined to be 100 and 79, respectively. The accuracy of the tumor grade (Figure 3A) and MVI (Figure 3B) nomogram model was favorable with an area under the ROC curve of 0.727 (95% CI: 0.690–0.761) and 0.839 (95% CI: 0.808–0.867), respectively. The sensitivity, specificity, positive predictive value, and negative predictive value when used in differentiating the presence from absence of tumor grade were 51.3, 81.05, 37.8, and 88.1%, and MVI were 80.46, 75.06, 55.3, and 90.9%, respectively (Table 4).

**Table 4:** Accuracy of the prediction score of the nomogram for estimating the risk of moderate/poor grade and MVI presence

Variable	Value (95% CI)	
	Moderate/poor grade	MVI
Sensitivity, %	51.30 (41.8–60.7)	80.46 (73.8–86.1)
Specificity, %	81.05 (77.4–84.4)	75.06 (70.8–79.0)
Positive predictive value, %	37.8 (30.2–45.9)	55.3 (49.0–61.6)
Negative predictive value, %	88.1 (84.8–90.9)	90.9 (87.5–93.6)
Positive likelihood ratio	2.71 (2.1–3.5)	3.23 (2.7–3.8)
Negative likelihood ratio	0.60 (0.5–0.7)	0.26 (0.2–0.4)
Youden Index, *J*	0.3236	0.5551
Area under ROC curve	0.727 (0.690–0.761)	0.839 (0.808–0.867)
Cutoff score	100	79

## Discussion

Surgical resection, transplantation, and ablation are therapies that provide a high rate of complete responses and, thus, potential for treatment of HCC. Resection remains the first choice for patients who have the optimal profile [25]. MVI, poor histological differentiation, satellites, and multifocal disease predict early recurrences after resection [26,27]. Lei et al. [28] have demonstrated MVI as an independent risk factor of tumor recurrence and overall survival (OS).

Attempts have been made in the past to identify relevant predictors of tumor grade and MVI. Data from Pawlik’s study suggested that tumor grade, preoperative serum AFP level, number of tumor nodules, and tumor size remained independent predictors of MVI in HCC patients [9]. Chen and co-workers demonstrated that the presence of ascites, a high tumor grade and AFP >1000 ng/ml were independently correlated with MVI by logistic regression analysis in patients with HBV-related cirrhotic HCC [29]. Non-smooth tumor margins on preoperative CT images were also a predictor of MVI in another research [30]. A work studied by Cucchetti et al. [31] developed an artificial neural network (ANN) model that predicted that preoperative serum AFP, tumor number, size, and volume were related to tumor grade and MVI. However, the wide use of this model is limited since it requires specific computer softwares. The nomogram provides a simple graphical representation of sophisticated statistical prediction factors and has been accepted as a reliable tool for predicting clinical matters [32]. Previously, a study from Lei et al. constructed nomograms to predict MVI in HBV-related HCC. However, other factors that have been recognized to be related to MVI were not included in the research.

It has been estimated that inflammation and chronic infections contribute to approximately 15% of all human cancers [33]. The development of HCC represents one of the inflammation-related carcinogenesis events since more than 90% of HCCs occur in the setting of hepatic injury and inflammation [34,35]. Previous studies have attempted to use serum and tumor biomarkers to predict MVI, but they did not focus on stage I/II HCC [6,28]. In the present study, we developed nomograms including inflammatory biomarkers to improve prediction of tumor grade and MVI in stage I/II HCC patients. Besides, our study suggested that tumor volume was significantly associated with tumor grade, and age as well as tumor size was linked with MVI.

Numerous studies have reported that inflammatory indices, such as NLR, LMR, and PLR are related to prognosis in patients with HCC [13,14,16,36–40]. Li et al. demonstrated patients with low NLR presented higher 6-month survival rate compared with patients with high NLR level in patients with advanced HCC [41]. A meta-analysis reached the same results that high NLR indicated a poor prognosis for patients with primary liver cancer and a high NLR was associated with the presence of tumor vascular invasion [42]. Results in Lin et al. [39] study revealed that a high preoperative LMR level was an independent predictor of OS and recurrence-free survival (RFS) in HBV-associated HCC patients after curative resection. Among the factors studied, poor histological differentiation was identified as an independent indicator for inferior RFS and OS, and MVI was an independent factor for OS. Another study demonstrated that high NLR and PLR were associated with poor prognosis in recurrent HCC treated with transarterial chemoembolization (TACE). But there were no significant differences in vascular invasion between the low and high groups for either NLR or PLR [36]. Few studies have indicated the relationship between dNLR and HCC. The results in the research of Zhou et al. [43] revealed that an elevated dNLR predicted a poor prognosis with a similar prognostic power to the NLR in patients with HBV-associated HCC undergoing TACE. In the present study, we found that pretreatment NLR and dNLR were independent prognostic factors of tumor grade, and dNLR and LMR were significant prognostic factors of MVI in patients with HCC. The mechanism of the prognostic value of these inflammatory biomarkers in cancer remains unclear. The inflammatory cells and the chemokines and cytokines that the tumor produced influence the whole tumor organ, regulate the growth, migration and differentiation of all cell types in the tumor micro-environment. The pro-tumor functions of inflammatory cells include releasing growth and survival factors, promoting angiogenesis and lymphangiogenesis, stimulating DNA damage, coating tumor cells to make available receptors for disseminating cells via lymphatics and capillaries, and evading host defense mechanisms [33]. A mounting evidence has shown that cytokines (IL-1, IL-6, IL-8, tumor necrosis factor-α), chemokines (CXCL12-CXCR4 axis, CX3CL1-CX3CR1 axis, and CCL20-CCR6 axis), and signaling pathways (NF-κB JAK-STAT3 EGFR signaling) are implicated in HCC [35,44]. These signaling molecules and pathways are interconnected with extensive crosstalk.

For clinical use of the model, we generalized the sensitivity, specificity, positive predictive value, and negative predictive value in estimating the risk of tumor grade and MVI using 100 and 79 as the cutoff value, respectively (Table 4). Patients with a score of 100 or more are a high-risk subgroup of poor grade. Similarly, patients with a score of 79 or more have a high risk of MVI. Based on these preoperative predictions, the nomogram might work as an instrument to select patients for randomized clinical trials for evaluating the efficacy of liver resection in patients with stage I/II HCC. Furthermore, it can provide guidance for choosing a more suitable treatment.

Undeniably, our study had several limitations. First, this analysis was based on data from a single center; it is necessary to validate the results from other institutions. Second, external validation is required to confirm the reliability of the nomogram using an independent dataset. Third, we only included patients with stage I and II in the present study. Thus, the results can not represent all patients with HCC. Fifth, our study was a retrospective study, in which the involving inflammatory markers were incomplete, such as C-reactive protein (CRP). Although there were several studies concerning the relationship between the prognosis of HCC and CRP [45,46], no study analyzed the relationship between CRP and liver cancer grading or micro-vascular infiltration. Nevertheless, CRP was detected in only 27 (4.3%) patients in our study. The same as CRP, HBV DNA level, which has been shown to be associated with prognosis of early-stage HCC and advanced HCC in previous studies [47–50] was also absent from our study. The association between HBV DNA and tumor grade or MVI should be validated in prospective studies. Some specific markers, such as serum osteopontin, serum endocan and serum VGEF were also indicated as good non-invasive biomarkers to predict different grades of HCC [51–53].

In conclusion, our study demonstrated that pretreatment serum inflammatory biomarkers were independent prognostic biomarkers for tumor grade and MVI in patients with stage I/II HCC. A nomogram was constructed depending on preoperative risk factors of tumor grade and MVI. The model provided an optimal preoperative estimation of tumor grade and MVI risk in patients with stage I/II HCC. It is hoped that the current nomograms can be further applied and validated in the clinical practices of other institutions.

## Supporting information

**Supplementary Table 1:** Etiology of the patients with hepatocellular carcinoma

**Supplementary Table 2:** Comparison of baseline characteristics based on tumor grade and micro-vascular invasion Presence.

## Abbreviations

BMI: body mass index
CI: confidence intervals
CRP: C-reactive protein
CT: computed tomography
dNLR: derived neutrophil-to-lymphocyte ratio
EGFR: epidermal growth factor receptor
HCC: hepatocellular carcinoma
IL: interleukin
JAK: Janus family kinase
LMR: lymphocyte-to-monocyte ratio
NF-κB: nuclear factor-κB
MVI: micro-vascular invasion
NLR: neutrophil-to-lymphocyte ratio
OR: odds ratio
OS: overall survival
PLR: platelet-to-lymphocyte ratio
ROC: receiver operating characteristic
RFS: recurrence-free survival
STAT: signal transducer and activator of transcription
TB: total bilirubin
