# Supplementary material for: Nomograms based on inflammatory biomarkers for predicting tumor grade and micro-vascular invasion in stage I/II hepatocellular carcinoma
Source: Biosci Rep. 2018 Nov 14;38(6):BSR20180464. doi: 10.1042/BSR20180464 (PMC6239277; doi:10.1042/BSR20180464)
Supplement: Supplementary file 1 [file bsr20180464_Supp1.pdf]

Supplementary Table 1. Etiology of the patients with hepatocellular carcinoma

Etiology (n,%)	All patients (n=627)
HBV	434 (69.2%)
Alcoholism	42 (6.7%)
HBV+ Alcoholism	116 (18.5%)
HCV	5 (0.8%)
Others	30 (4.8%)

HBV, hepatitis B virus; HCV, hepatitis C virus;

Supplementary Table 2. Comparison of baseline characteristics based on tumor grade and micro-vascular invasion Presence.

	Tumor grade		P	MVI		P
	1/2	3/4		Presence	Absence	
Age (year)	55.99±11.37	56.04±12.54	0.967	53.58±11.81	56.93±11.37	0.001
BMI(kg/m2)	22.35±3.80	23.07±4.07	0.069	22.65±3.73	22.41±3.91	0.50
Male sex - n (%)	432(84.38%)	100(86.96%)	0.485	149(85.63%)	383(84.55%)	0.734
Neutrophil (*10^9/L)	3.04±1.28	4.24±1.92	<0.001	3.92±1.83	3.01±1.25	<0.001
Lymphocyte (*10^9/L)	1.85±0.79	1.85±0.93	0.980	1.81±0.83	1.86±0.81	0.522
Monocyte (*10^9/L)	0.57±0.32	0.60±0.28	0.373	0.89±0.34	0.45±0.20	<0.001
NLR	1.88±1.20	3.28±3.03	<0.001	2.74±2.41	1.91±1.39	<0.001
LMR	4.33±3.75	3.74±2.47	0.109	2.28±1.44	4.97±3.84	<0.001
PLR	87.46±71.66	108.64±91.88	0.022	97.72±92.75	88.90±68.65	0.194
dNLR	2.38±4.41	6.03±13.18	0.004	5.94±11.68	1.94±3.46	<0.001
TB (umol/L)	1.12±0.25	1.16±0.26	0.090	1.18±0.27	1.11±0.24	0.002
Albumin (g/L)	38.02±4.79	38.18±5.28	0.752	38.24±6.38	37.97±4.16	0.609
ALT (u/L)	54.44±65.40	51.60±49.72	0.661	55.95±53.22	53.14±66.14	0.616
AST (u/L)	63.95±113.61	59.77±67.41	0.726	70.65±79.32	60.53±114.30	0.344
AKP (u/L)	104.96±88.67	106.69±49.68	0.859	104.23±60.40	105.67±88.68	0.872
GGT (u/L)	96.33±155.30	101.63±99.55	0.758	97.86±113.13	97.22±155.56	0.968
Creatinine (mg/dl)	0.78±0.17	0.76±0.19	0.164	0.76±0.18	0.78±0.18	0.316
PT (s)	14.31±2.08	14.21±1.21	0.603	14.27±1.53	14.30±2.08	0.883
PTA (%)	87.26±14.75	87.95±13.16	0.643	86.81±14.41	87.61±14.49	0.538
INR	1.12±0.25	1.95±9.04	0.330	1.12±0.16	1.33±4.58	0.537

WB C(*10^9/L)	5.77±2.76	6.95±9.00	0.013	6.62±7.59	5.74±2.66	0.031
Platelets (*10^9/L)	135.19±70.53	144.84±64.95	0.180	138.31±77.19	136.45±66.52	0.765
lg AFP (ng/mL)	1.93±1.09	2.07±1.15	0.223	2.16±1.12	1.87±1.09	0.004
tumor size	4.76±3.18	6.29±3.72	<0.001	5.96±3.91	4.69±3.01	<0.001
tumor volume (Log10 cm3)	1.42±0.80	1.83±0.91	<0.001	1.65±0.89	1.44±0.81	0.005
Tumor number (n,%)			0.155			0.561
1	433(84.57%)	91(79.13%)		143(82.18%)	381(84.11%)	
>1	79(15.43%)	24(20.87%)		31(17.82%)	72(15.89%)	
Cirrhosis (n,%)			0.877			0.156
yes	330(64.45%)	75(65.22%)		120(68.97%)	285(62.91%)	
no	182(35.55%)	40(34.78%)		54(31.03%)	168(37.09%)	

BMI, body mass index; NLR, neutrophil-to-lymphocyte ratio; LMR, lymphocyte-to-monocyte ratio; PLR, platelet-to-lymphocyte ratio; dNLR, neutrophil-to -(white cell count - neutrophil count); TB, total bilirubin; ALT, alanine aminotransferase; AST, aspartate aminotransferase; AKP, alkline phosphatase; GGT, γ-glutamyltransferase; PT, prothrombin time; PTA, prothrombin activity; INR, international normalized ratio; WBCs, white blood cells; HBV, hepatitis B virus; MVI, micro-vascular invasion.
